# Molecular Epidemiological Analysis of Carbapenem-Resistant Hypervirulent Klebsiella Pneumoniae in Cangzhou Central Hospital from 2021 to 2024

**DOI:** 10.12669/pjms.42.7.13601

**Published:** 2026-07

**Authors:** Li Xu, Lan Chen, Yan Sun, Yuezhen An

**Affiliations:** 1 Li Xu, Department of Laboratory Medicine, Cangzhou Central Hospital, Cangzhou 061000, Hebei, China; 2 Lan Chen, Department of Laboratory Medicine, Cangzhou Central Hospital, Cangzhou 061000, Hebei, China; 3 Yan Sun, Department of Laboratory Medicine, Cangzhou Central Hospital, Cangzhou 061000, Hebei, China; 4 Yuezhen An, Department of Laboratory Medicine, Cangzhou Central Hospital, Cangzhou 061000, Hebei, China

**Keywords:** Carbapenem-Resistant Hypervirulent Klebsiella Pneumoniae, Epidemiology, Drug Resistance

## Abstract

**Objective:**

To investigate the molecular epidemiology of carbapenem-resistant hypervirulent Klebsiella pneumoniae (CR-hvKP) in Cangzhou Central Hospital from 2021 to 2024.

**Methodology:**

This was a retrospective study. A total of one hundred patients clinically diagnosed with CR-hvKP in Cangzhou Central Hospital from June 2021 to December 2024 were selected and divided into a survival group and a mortality group based on mortality within 30 days. Afterward, clinical data were collected, with Logistic analyses utilized to identify factors influencing mortality in CR-hvKP patients.

**Results:**

The majority of CR-hvKP patients were found in the neurology department(23.00%) and the intensive care unit (ICU)(20.00%). The 100 strains of CR-hvKP primarily originated from sputum(29.00%) and wound secretion(20.00%) samples. Meanwhile, the main types of infections among the one hundred patients included respiratory(57.00%), bloodstream(13.00%), and intracranial (13.00%) infections. Additionally, CR-hvKP isolates were highly resistant to most antibiotics, except for being susceptible to colistin and tigecycline. Moreover, significant differences were observed between the survival and death groups in terms of diabetes, ICU history, mechanical ventilation, and tigecycline(*P*< 0.05). Furthermore, Logistic analyses indicated diabetes, ICU history, and mechanical ventilation as risk factors for mortality in CR-hvKP patients(*P*< 0.05) and tigecycline as a protective factor(*P*< 0.05).

**Conclusion:**

Factors influencing mortality in CR-hvKP patients included diabetes, ICU history, mechanical ventilation, and tigecycline. Therefore, preventive measures could be developed based on relevant factors to reduce patient mortality.

## INTRODUCTION

Klebsiella pneumoniae (KP), a pathogenic bacterium that causes nosocomial infections, can colonize the mucosal surfaces of the mouth, nose, and gastrointestinal tract, leading to wound infections, bloodstream infections, etc.^1^ In recent years, the drug resistance of KP has gradually increased. According to bacterial resistance results in 2021, KP showed significantly higher resistance to carbapenems compared to that before 2018, which, despite a decreasing trend in recent years, still exceeds 20%, indicating challenges in most antibiotic treatments for KP.^2^ Based on its virulence characteristics, KP can be classified into the classical KP and hypervirulent KP(hvKP), with the latter being a highly invasive variant of KP that can cause purulent abscesses, infect distal host tissues, and lead to infections in multiple sites, ultimately triggering severe and critical conditions and even mortality.^3^

In recent years, the emergence of carbapenemase-producing strains has led to the widespread transmission of carbapenem-resistant KP(CRKP), which carries resistance genes such as blaKPC and limits the options for antimicrobial agents, thus increasing treatment difficulties.^4^ In this regard, hypervirulent CRKP (CR-hvKP) is characterized by high pathogenicity and resistance, making it a major risk factor for mortality in nosocomial infections, posing challenges for clinical treatment.^5^ Currently, studies exploring the epidemiology and drug resistance of CR-hvKP remain limited, with no analysis available on factors affecting the prognosis of CR-hvKP-infected patients. Therefore, this study is designed to investigate the molecular epidemiology of CR-hvKP in Cangzhou Central Hospital from 2021 to 2024.

## METHODOLOGY

This was a retrospective study. According to the actual situation of the hospital and statistical estimation. A total of one hundred patients clinically diagnosed with CR-hvKP in Cangzhou Central Hospital from June 2021 to December 2024 were randomly selected.

### Ethical Approval:

The study was approved by the Institutional Ethics Committee of Cangzhou Central Hospital (No.:2021-079-01; Date: June 04, 2021) and written informed consent was obtained from all participants.

### Inclusion criteria:

- Patients who met the criteria for CR-hvKP^6^ and were confirmed by microbiological examination
- Age > 18 years.
- Strains were isolated upon the patient’s first admission.

### Exclusion criteria:

- Patients with repeated strains isolated from the same site.
- Patients with incomplete clinical data.
- Patients with mental disorders.

### • Diagnostic Criteria for CR-hvKP:^6^

- Samples should be sterile body fluids (e.g., blood, effusion, and cerebrospinal fluid) and could be diagnosed when microbiological testing is positive;
- For samples such as sputum, various swabs, wound secretions, and feces, diagnosis could be confirmed when identical CR-hvKP strains (≥ 2) were cultured from the same sample;
- If only one strain was cultured, diagnosis could be confirmed by combining with clinical symptoms: Body temperature > 38°C or < 36°C;, Dyspnea and chills; and presence of purulent secretions;
- Imaging findings showing signs of pleural effusion, abscesses, etc.

Identification and Antimicrobial Susceptibility Testing of CR-hvKP Strains: Strains were identified using the Bruker Microflex LT MALDI-TOF (quality control strains included Escherichia coli ATCC25922 and Klebsiella pneumoniae ATCC700603). Results were interpreted following relevant standards,^7^ requiring that the minimum inhibitory concentration (MIC) of imipenem be ≥16 μg/mL, and that the string test and virulence gene rmp4 be positive to classify the strain as CR-hvKP. Meanwhile, antimicrobial susceptibility was determined using the BD Phoenix M50 Automated Microbiology System, following relevant standards.^8^

### Follow-Up:

Follow-up began on the day of positive CR-hvKP cultured in the patients, with all-cause mortality as the endpoint (within 30 d). Patients who improved and were discharged early were still followed up to 30 d (via phone or WeChat) and then were divided into the survival and death groups based on the clinical outcomes.

### Observation Indicators and Collection of Clinical Data:

- Analysis of the departments where CR-hvKP patients were infected;
- Statistics on the sources of isolates from CR-hvKP patients;
- Statistics on the types of infections in CR-hvKP patients;
- Analysis of the drug resistance of CR-hvKP isolates;
- Comparison of clinical data of the survival and death groups (age, gender, BMI, diabetes, respiratory diseases, digestive diseases, neurological diseases, liver and kidney diseases, cardiovascular diseases, malignant tumors, cerebrovascular diseases, length of hospital stay, ICU history, mechanical ventilation, surgery, endotracheal tubes, central venous catheters, urinary catheters, nasogastric tubes, blood purification, drainage tubes, tigecycline);
- Multivariate analysis of factors influencing mortality in CR-hvKP patients.

### Statistical Analysis:

SPSS 25.0 was used for data processing. Count data were analyzed using the *χ²* test and expressed as n (%), while measurement data were analyzed using the t-test and expressed as (). Factors influencing mortality in CR-hvKP patients were investigated through the logistic analysis. P<0.05 was considered statistically significant.

## RESULTS

Statistical analysis suggested no statistical differences in baseline data between the two groups, as shown in Table-I.

**Table-I:** Department of CR-hvKP-Infected Patients.

Department	Number (n = 100)	Proportion
Neurology	23	23.00
Neurosurgery	16	16.00
Hematology	15	15.00
ICU	20	20.00
Nephrology	7	7.00
Respiratory Medicine	6	6.00
General Surgery	4	4.00
Urology	4	4.00
Oncology	3	3.00
Emergency Medicine	2	2.00

The departments of CR-hvKP patients were mainly the neurology department (23.00%) and the ICU (20.00%) Table-I. A total of 100 CR-hvKP strains were mainly from sputum (29.00%) and wound secretion (20.00%) samples Table-II.

**Table-II:** Sample Sources for Isolates from CR-hvKP Patients.

Sample Type	Number (n = 100)	Proportion
Sputum	29	29.00
Blood	12	12.00
Wound Secretion	20	20.00
Buccal Swab	9	9.00
Throat Swab	9	9.00
Drainage Fluid	14	14.00
Urine	2	2.00
Feces	5	5.00

The types of infections in the one hundred patients mainly included respiratory system (57.00%), bloodstream (13.00%), and intracranial (13.00%) infections, as shown in Table-III. CR-hvKP isolates were highly resistant to other antibiotics, except for being susceptible to colistin and tigecycline Table-IV. Differences were observed in diabetes, ICU history, mechanical ventilation, and tigecycline between the survival and death groups(*P* < 0.05), with no differences in the other general data (*P* > 0.05) Table-V.

**Table-III:** Types of Infections in CR-hvKP Patients.

Infection Type	Number (n = 100)	Proportion
Respiratory System Infection	57	57.00
Bloodstream Infection	13	13.00
Intracranial Infection	13	13.00
Abdominal Infection	5	5.00
Urinary Tract Infection	2	2.00
Others	10	10.00

**Table-IV:** Drug Resistance of CR-hvKP Isolates.

Antibiotic	Drug Resistance		Mediator		Sensitivity	
	Strain (n)	Resistance Rate (%)	Strain (n)	Mediation Rate (%)	Susceptible (strains)	Susceptibility (%)
Cefotaxime	100	100.00	0	0.00	0	0.00
Cefepime	100	100.00	0	0.00	0	0.00
Ceftazidime	100	100.00	0	0.00	0	0.00
Cefmetazole	100	100.00	0	0.00	0	0.00
Ceftriaxone	100	100.00	0	0.00	0	0.00
Cefuroxime	100	100.00	0	0.00	0	0.00
Ciprofloxacin	100	100.00	0	0.00	0	0.00
Imipenem	100	100.00	0	0.00	0	0.00
Meropenem	100	100.00	0	0.00	0	0.00
Ertapenem	100	100.00	0	0.00	0	0.00
Ampicillin	100	100.00	0	0.00	0	0.00
Ampicillin/Sulbactam	100	100.00	0	0.00	0	0.00
Cefoperazone/Sulbactam	100	100.00	0	0.00	0	0.00
Aztreonam	100	100.00	0	0.00	0	0.00
Colimycin	8	8.00	12	12.00	80	80.00
Amikacin	82	82.00	10	10.00	8	8.00
Tigecycline	15	15.00	12	12.00	73	73.00
Gentamicin	73	73.00	17	17.00	10	10.00
Ciprofloxacin	86	86.00	8	8.00	6	6.00
Trimethoprim-Sulfamethoxazole	46	46.00	20	20.00	34	34.00

**Table-V:** Comparison of Clinical Data between Patients in the Survival and Death Groups.

Group	Survival Group (n=59)	Death Group (n=41)	t/χ^2^	P
Age (Years)	53.69±7.59	55.26±8.81	0.952	0.343
M/F	38/21	29/12	0.438	0.508
BMI (kg/m^2^)	23.25±2.37	23.19±2.43	0.123	0.902
Diabetes	13(22.03)	20(48.78)	7.827	0.005
Respiratory Diseases	7(11.86)	10(24.39)	2.690	0.101
Digestive Diseases	19(32.20)	15(36.59)	0.207	0.649
Nervous System Diseases	18(30.51)	14(34.15)	0.147	0.701
Liver and Kidney Diseases	5(8.47)	5(12.20)	0.372	0.542
Cardiovascular Diseases	14(23.73)	14(34.15)	1.302	0.254
Malignant Tumor	8(13.56)	7(17.07)	0.234	0.628
Cerebrovascular Diseases	9(15.25)	10(24.39)	1.312	0.252
Hospital Stay (d)	9.34±2.15	9.43±2.23	0.379	0.706
ICU History	14(23.73)	21(51.22)	8.036	0.005
Mechanical Ventilation	24(40.68)	28(68.29)	7.390	0.007
Surgery	14(23.73)	13(31.71)	0.781	0.377
Endotracheal Tube	16(27.12)	15(36.59)	1.014	0.314
Central Venous Catheter	9(15.25)	10(24.39)	1.312	0.252
Urinary Catheter	14(23.73)	13(31.71)	0.781	0.377
Nasogastric Tube	9(15.25)	12(29.27)	2.864	0.091
Blood Purification	10(16.95)	11(26.83)	1.423	0.233
Drainage Tube	15(25.42)	15(36.59)	1.435	0.231
Tigecycline	19(32.20)	3(7.32)	8.731	0.003

The Logistic (stepwise forward) analysis was conducted with the dependent variable as whether CR-hvKP patients died (yes = 1, no = 0) and the independent variable as the indicator of the above differences (diabetes = 1, no = 0; ICU history = 1, no = 0, mechanical ventilation = 1, no = 0, tigecycline = 1, no= 0), suggesting that diabetes, ICU history, and mechanical ventilation were risk factors influencing mortality in CR-hvKP patients(*P*< 0.05) and tigecycline was a protective factor (*P* < 0.05) Table-VI.

**Table-VI:** Multivariate Analysis of Factors Influencing Mortality in CR-hvKP Patients.

Indicator	B	SE	Waldχ^2^	P	OR	95% CI
Diabetes	1.114	0.415	7.212	0.007	3.048	1.351~6.875
ICU History	0.743	0.312	5.677	0.017	2.103	1.141-3.876
Mechanical Ventilation	1.133	0.402	7.939	0.005	3.104	1.412-6.825
Tigecycline	-0.669	0.304	4.849	0.028	0.512	0.282~0.929

## DISCUSSION

The findings of this study indicate that CR-hvKP patients primarily come from the neurology department and the ICU, as those in these two departments often develop more severe conditions and are accompanied by various underlying diseases, leading to poor immune function and increased susceptibility to bacterial colonization and subsequent infections.^9^,^10^ In this regard, clinical management of CR-hvKP infections should be strengthened in these relevant departments. In the meantime, CR-hvKP strains are mainly isolated from sputum, as mechanical ventilation and other interventions can damage the respiratory mucosal barrier, making it difficult for patients to expectorate, which facilitates CR-hvKP invasion and ultimately leads to respiratory infections.^11^ Therefore, in clinical practice, strict control of the timing and procedures for invasive operations is necessary, along with prompt assessments of patient conditions to wean as early as possible, so as to reduce infection risks.

Additionally, respiratory infections are the primary type associated with CR-hvKP and potentially related to the invasive procedures patients undergo. Classified by the World Health Organization as one of the six major multidrug-resistant bacteria, hvKP is a major infection-inducing pathogenic bacterium, the resistance rate of which to imipenem and meropenem exceeds 20% according to the 2023 CHINET survey, severely limiting the options and efficacy of clinical drugs, thus significantly increasing patient mortality.^12^ CR-hvKP has been identified in multiple countries, including China and the United States, and is particularly prevalent in East China. Notably, the extensive use of antibiotics has led to the development of resistance in KP strains, and the presence of hypervirulent genes and resistance genes on the same plasmid has resulted in CR-hvKP, which not only exhibits high virulence but also leads to severe invasive infections. Additionally, due to its high antibiotic resistance, there is a lack of effective clinical treatment options, resulting in an increasing mortality in patients.^13^ Moreover, this study also found that CR-hvKP isolates, except for being susceptible to colistin and tigecycline, are highly resistant to most antibiotics, suggesting that colistin and tigecycline could be used clinically to treat patients and improve their conditions.

The Logistic analysis in this study identified diabetes, ICU history, mechanical ventilation, and tigecycline as factors influencing mortality in CR-hvKP patients, with analyses of these factors as follows:

Hyperglycemia is conducive to bacterial growth, which increases the glycosylation and excretion of albumin when drugs transport proteins, reducing drug utilization and leading to poor prognosis. Severe infections also cause stress-induced hyperglycemia, elevating blood sugar levels in hospitalized patients. Inflammatory infections act as stimuli that increase cortisol secretion and insulin resistance, further raising blood sugar levels, making diabetic patients more susceptible to infections.^14^ Therefore, it is important to monitor blood sugar levels in patients and provide treatment when necessary.

The mortality in ICU patients is higher, consistent with previous studies,^15^ as these patients often require multiple rounds of antibiotic treatments, leading to multidrug resistance. Specifically, approximately 35% of patients receive empirical treatment during hospitalization, which results in resistance to hvKP, limiting drug options and affecting treatment outcomes, thereby increasing mortality.^16^ In the meantime, these patients also tend to experience compromised immune function and underlying diseases, making them more susceptible to CR-hvKP infections during invasive treatments, ultimately resulting in poor prognosis and increased mortality.^17^

Patients who have undergone invasive procedures such as mechanical ventilation exhibit significantly lower survival rates. Such procedures are often emergency measures for critically ill patients and can disrupt the respiratory mucosal barrier, allowing bacteria to invade and proliferate.^18^ Studies have shown that over half of infection cases are related to invasive procedures, which can be considered a triggering factor for patient mortality.^19^ Therefore, it is essential to closely monitor changes in patient conditions and avoid prolonged ventilation therapy.

Tigecycline, a restrictive antimicrobial agent, is highly susceptible to CR-hvKP and can serve as a protective factor for the prognosis of patients, which can prevent transfer RNA from entering the ribosome and inhibit bacterial protein synthesis, thereby exhibiting antibacterial effects^4^. While tigecycline is the preferred treatment for CR-hvKP, a rising trend in resistance rates has been reported in recent years with the increase in its usage.^20^ Therefore, susceptibility testing should be conducted when using this drug clinically, and treatment should be administered based on susceptibility results, so as to alleviate resistance, prolong its effective use as much as possible, and prevent the emergence of drug-resistant bacteria.

### Limitations

It includes a small sample size and being a single-center study, which may affect the representativeness of its findings. Therefore, a larger sample size and multicenter studies are required in the future for the validation of the findings.

## CONCLUSIONS

In conclusion, the neurology department was the primary source of CR-hvKP infections at Cangzhou Central Hospital from 2021 to 2024, with respiratory infections being the most common ones and susceptible to colistin and tigecycline. Factors influencing mortality in CR-hvKP patients included diabetes, ICU history, mechanical ventilation, and tigecycline. In this regard, preventive measures should be developed based on relevant factors to reduce patient mortality.

### Authors’ Contributions:

**LX:** Designed, did statistical analysis & editing of manuscript, is responsible for integrity of research.

**LC:** Collected and analyzed clinical data. Critical Review.

**YS** and **YA:** Literature search**,** significantly revised this manuscript.

All authors have read and approved the final manuscript.
